# Diagnostic Performance of Vessel Wall Magnetic Resonance Imaging (VW-MRI) for Intracranial Vasculopathies: A Systematic Review and Meta-Analysis

**DOI:** 10.7759/cureus.97296

**Published:** 2025-11-19

**Authors:** Mohammed F Albalawi, Abdulmajeed Albalawi, Eslam M Rabea, Mohammed Alqahtani, Fawaz Alotaibi, Muhannad Asiri, Sawsan Bakry, Hussain BinAmir, Samar Emad Eshetawe, Alaa Heji, Hossam M Ghorab, Fahad Alajlan, Ashfaq Shuaib, Abdulrahman Ayidh Alrasheed, Adel Alhazzani

**Affiliations:** 1 Department of Neurology, King Faisal Specialist Hospital and Research Centre, Riyadh, SAU; 2 Department of Internal Medicine, Ministry of National Guard Health Affairs, Tabuk, SAU; 3 Department of Neurosurgery, Alexandria Main University Hospital, Alexandria, EGY; 4 Department of Neurology, King Fahad General Hospital, Jeddah, SAU; 5 Department of Medicine, Armed Forces Hospital - Southern Region, Khamis Mushait, SAU; 6 Department of Internal Medicine, Alexandria University Faculty of Medicine, Alexandria, EGY; 7 Department of Neurology, University of Alberta, Alberta, CAN; 8 Department of Neurology, Prince Sultan Military Medical City, Riyadh, SAU

**Keywords:** intracranial, sensitivity, specificity, vasculopathies, vessel wall mri

## Abstract

Vessel wall magnetic resonance imaging (VW-MRI) provides superior capabilities, compared to traditional angiography, in evaluating intracranial vessel walls. This study aimed to assess the diagnostic performance of VW-MRI in intracranial vasculopathies.

This systematic review and meta-analysis followed PRISMA guidelines. PubMed, Scopus, Web of Science, Cochrane Library, and ScienceDirect were searched to identify studies investigating the diagnostic performance of VW-MRI in intracranial vasculopathies. Eligible studies should report sufficient data to construct 2 × 2 contingency tables. Disease categories were included only if at least two studies were available. Pooled sensitivity, specificity, diagnostic odds ratio (DOR), and area under the curve (AUC) were estimated using a bivariate model. Study quality was assessed using the QUADAS (Quality Assessment of Diagnostic Accuracy Studies) tool.

Ten studies on intracranial atherosclerotic disease (ICAD) demonstrated high diagnostic accuracy (sensitivity 0.877; specificity 0.808; DOR 34.2; AUC 0.907). Subgroup analyses confirmed reliability in detecting stenosis, distinguishing culprit from non-culprit lesions, and identifying eccentric wall thickening as a biomarker. Six studies on intracranial vasculitis showed strong diagnostic performance of concentric wall enhancement (sensitivity 0.817; specificity 0.864; DOR 44.9; AUC 0.909). Two studies on intracranial dissection reported high accuracy (sensitivity 0.901; specificity 0.829; DOR 58.1; AUC 0.930).

VW-MRI demonstrates excellent diagnostic performance for ICAD, intracranial vasculitis, and dissection. It provides added value beyond conventional imaging by enabling evaluation of vessel wall features and lesion characterization. Further high-quality studies, with larger sample sizes, are needed to validate its clinical utility.

## Introduction and background

Intracranial atherosclerotic disease (ICAD) is a common cause of ischemic stroke worldwide, accounting for 10% of all ischemic strokes in the United States and 50% in Asia [1-2]. However, the true prevalence of ICAD remains uncertain and varies widely across studies due to methodological heterogeneity, differences in study populations, and the lack of standardized definitions and imaging protocols for diagnosing ICAD and intracranial atherosclerotic stenosis (ICAS) [3]. Furthermore, current diagnostic tools may overlook many non-stenotic or early-stage lesions, potentially underestimating the actual burden of the disease. In contrast to extracranial carotid disease, ICAD remains not fully characterized due to the small caliber, tortuous pathways, and deep location of intracranial arteries within the skull. Additionally, obtaining tissue samples from patients with ICAD is challenging, making it difficult to conduct pathology-imaging correlation studies. Consequently, active research on ICAD is limited [4]. Therefore, the development of advanced intracranial vascular imaging techniques is essential for enhancing our understanding and management of intracranial vasculopathies as a whole. While ICAD represents the most common etiology, intracranial vasculopathies encompass a broader spectrum of conditions beyond atherosclerosis, including vasculitis, dissection, moyamoya disease, vasospasm, fibromuscular dysplasia, and aneurysms. To encompass these diverse pathologies, the term “nonatherosclerotic intracranial large artery disease” has been introduced [5]. Improved imaging modalities are therefore crucial for differentiating among these entities, guiding tailored therapeutic approaches, and improving outcomes.

Luminal evaluation using conventional techniques, such as catheter angiography, computed tomography angiography (CTA), digital subtraction angiography (DSA), and magnetic resonance angiography (MRA), cannot characterize different pathologies within the vessel wall. Therefore, some cases of ICAD were misclassified as atherosclerotic. Intracranial vasculopathy requires completely different management approaches, and treatment delays or inappropriate therapy can cause significant morbidity and even mortality [5-6].

Strategies for visualization of the vascular walls of intracranial arteries using high-resolution vessel wall magnetic resonance imaging (VW-MRI) have been developed to overcome these challenges. The principal technical requirements for intracranial VW-MRI are high spatial resolution (sub-millimetric spatial resolution), multiplanar 2D or 3D acquisitions, multiple tissue weightings, and luminal blood and cerebrospinal fluid signal suppression. VW-MRI enables differentiating intracranial vasculopathies through the analysis of multiple vessel wall characteristics, such as the presence of contrast enhancement; the pattern of wall involvement (eccentric vs. circumferential); the pattern of enhancement; the presence of remodeling; and T2 signal characteristics. Additionally, it helps evaluate nonstenotic lesions and characterize them by assessing atherosclerotic plaque activity, vasculitis activity, and risk of aneurysm rupture [7-9]. Several studies have assessed the diagnostic accuracy of VW-MRI for different ICAD. This meta-analysis aimed to quantitatively synthesize the existing evidence on the diagnostic power of VW-MRI for intracranial atherosclerosis, vasculitis, and dissection.

## Review

Methods

This meta-analysis was performed in accordance with the PRISMA and Cochrane handbook guidelines [10,11].

Databases and Search Terms

A systematic search was performed on PubMed, Scopus, WOS, Cochrane Library, and ScienceDirect until February 2024, without any date or language restrictions, using the following search terms: MRI vessel wall, magnetic resonance imaging vessel wall, MR vessel wall, vessel wall MRI, VW-MRI, vessel wall imaging, VWI, vasculopathy, vascular diseases, atherosclerosis, vasculitis, arteritis, and dissection. This search was supported by manual screening throughout the study period.

Eligibility Criteria

Primary studies investigating the diagnostic accuracy of VW-MRI for intracranial atherosclerosis, vasculitis, or dissection were eligible for inclusion. Eligible studies should include data on the frequencies of true positives (TP), false positives (FP), true negatives (TN), and false negatives (FN). At least two studies per disease were required for inclusion. No constraints were placed on the reference standard modality. Given the absence of a universally accepted gold standard for diagnosing intracranial vasculopathies, since brain or vessel wall biopsy is rarely feasible or ethical, we adopted the clinicoradiological diagnosis as the reference standard. This definition, used by most included studies, integrated clinical presentation and conventional imaging findings (DSA, CTA, or MRA) to establish the final diagnosis. Imaging modalities were therefore considered components of the diagnostic framework rather than independent reference tests. Case reports, comments, letters, animal experiments, review studies, original studies with incomplete data to form a two-by-two contingency table, and studies assessing extracranial rather than intracranial vasculopathy were excluded.

Study Selection and Data Extraction

The studies were screened for relevance by two independent reviewers. In case of any disagreement, consensus was reached through discussion with a third reviewer. The full texts of the potentially eligible studies were obtained and screened. The included studies were read carefully to collect relevant data into Microsoft Excel spreadsheets (Microsoft® Corp., Redmond, WA, USA). The summary data of the included studies were tabulated, including the first author’s name, publication year, sample size, number of lesions, mean age of participants, study design, study question, imaging protocol, and reference standard modality.

The diagnostic parameters of VW-MRI, including TP, TN, FN, and FP, were extracted or calculated from the sensitivity and specificity parameters. In case the study reported both 2D and 3D VW-MRI data, only 3D VW-MRI data were extracted. The primary outcomes of interest were the pooled diagnostic accuracy metrics, including sensitivity, specificity, diagnostic odds ratio (DOR), and area under the curve (AUC).

Data Synthesis and Analysis

From each study, we pulled the 2 × 2 numbers (TP, FP, FN, and TN). No cells were missing. We listed study-level sensitivity and specificity in a table and showed them in forest plots. To pool accuracy, we used a bivariate random-effects model [12]. This model estimates sensitivity and specificity together and allows for their usual trade-off when thresholds differ (the “threshold effect”), which is preferable to pooling them separately [13]. We also drew summary receiver operating characteristic (SROC) curves showing each study and the model’s summary point with 95% CIs. We explored heterogeneity with bivariate meta-regression by outcome definition. Among the 10 VW-MRI atherosclerosis studies, outcomes were: culprit vs. non-culprit lesions (n = 4), detection of >50% stenosis (n = 3), and eccentric wall thickening (n = 2). One study reported luminal stenosis detection only; it stayed in the overall analysis but was not used for subgroup modeling. Tools for publication-bias checks in bivariate diagnostic-accuracy meta-analyses are still limited. As a practical sensitivity check, we applied a left-sided trim-and-fill on the funnel of the log DOR [14,15] to see how potentially missing studies might influence results. All analyses were done in R (R Foundation for Statistical Computing, Vienna, Austria) using mada for the bivariate model and SROC estimation [16,17], and metafor for trim-and-fill [18].

Quality Assessment

The quality of each included study was assessed by two independent reviewers using the Quality Assessment of Diagnostic Accuracy Studies (QUADAS) tool. The QUADAS tool comprises 14 questions. Each question is answered with “yes,” “no,” or “unclear,” with “yes” being scored 1, and “unclear” or “no” being scored 0 [19].

Results

Search Results

**Figure 1:**
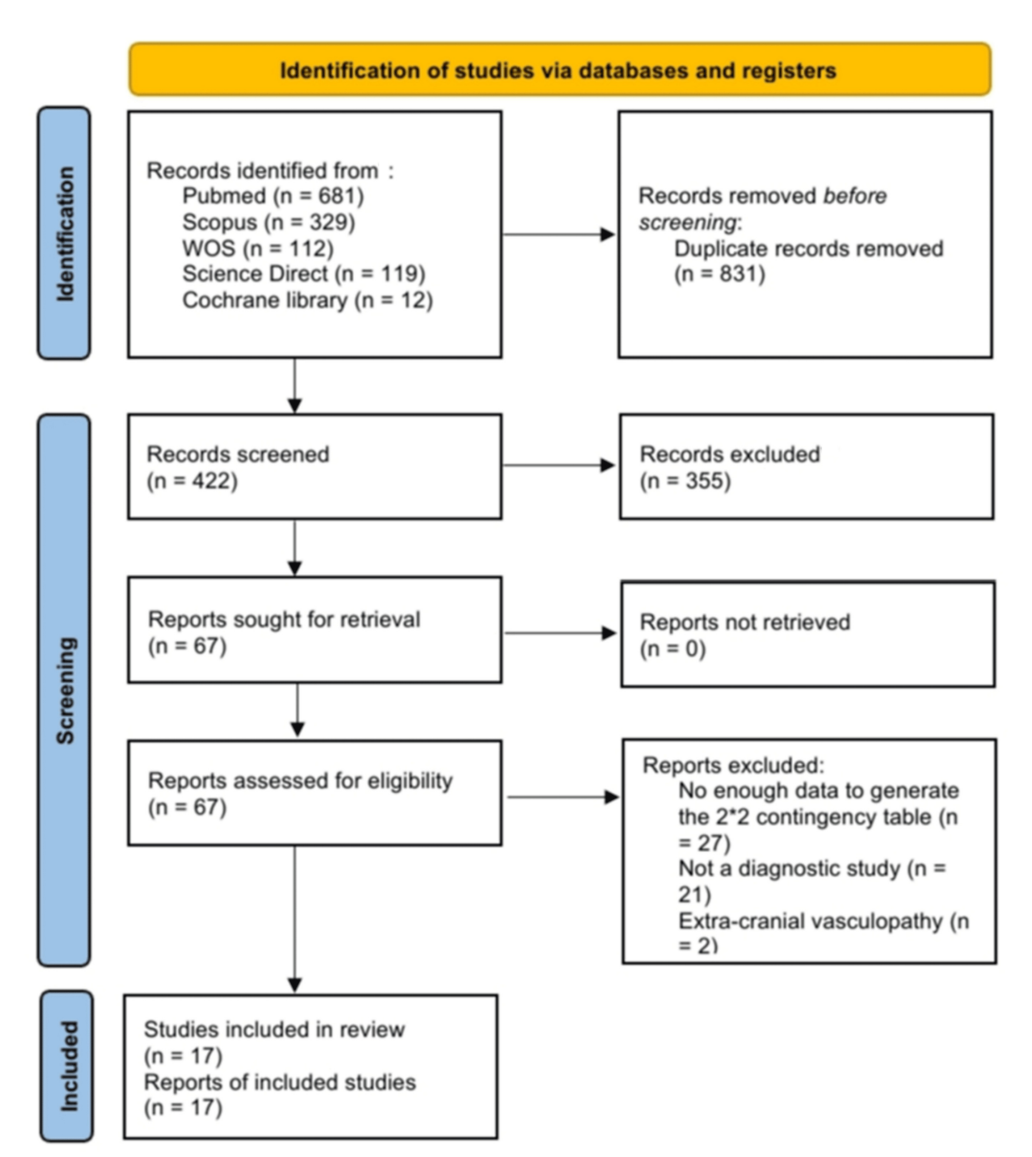
PRISMA flowchart

A total of 1,253 records were retrieved from the databases. After exclusion of duplicates, a total of 422 records were screened for relevance. The full texts of 67 studies were screened for meeting our prespecified eligibility criteria. Among them, 17 studies were included in this meta-analysis [20-36]. Figure 1 shows the search results.

Study Characteristics

Overall, 17 studies with a total of 893 patients were included. All included studies are observational: nine studies are retrospective, seven studies are prospective, and one of them is a cross-sectional study. Ten studies reported on diagnostic parameters of VW-MRI in intracranial atherosclerosis. Out of these 10 studies, four studies focused on the diagnostic power of VW-MRI in differentiating culprit plaques vs. non-culprit plaques in cases with cerebrovascular accidents [25,27-29], two studies investigated the performance of eccentric wall thickening (with or without other biomarkers, such as T2 hyperintense lesions) as a radiologic biomarker for intracranial atherosclerosis [20,21], and four studies reported on the diagnostic power of VW-MRI in the detection of luminal stenosis [22-24,26].

The studies focusing on differentiating culprit vs. non-culprit plaques used variable imaging parameters, such as enhancement pattern, degree of stenosis, plaque burden (PB), plaque volume (PV), plaque length (PL), minimum luminal area (MLA), site of origin involvement, and surface irregularity, which may contribute to heterogeneity in their reported diagnostic performance. Bai et al. [25] and Fakih et al. [28] used 7-T VW-MRI. Six studies investigated the diagnostic performance of concentric vessel wall enhancement in the diagnosis of intracranial vasculitis [21,32-36]. Out of the 17 included studies, two studies focused on the diagnostic performance of VW-MRI in intracranial dissections [30,31].

There are notable differences in the reference standard modalities used in each study. However, most of them depend on a clinico-radiologic diagnosis. Some studies compared the findings of VW-MRI with DSA, CTA, or MRA findings. Table 1 shows the characteristics of the included studies.

**Table 1:** Summary of the included studies IVWI, Intracranial vessel wall imaging; TOF, Time of flight; VBDA, Vertebrobasilar dissecting aneurysm; ICAD, Intracranial atherosclerotic disease; RCVS, Reversible cerebral vasoconstriction syndrome; VBDA, Vertebrobasilar dissecting aneurysm; HR-MRI, High-resolution magnetic resonance imaging; 3D, Three-dimensional; 2D, Two-dimensional; CE, Contrast-enhanced; MCA, Middle cerebral artery; ACA, Anterior cerebral artery; ICA, Internal carotid artery; VW-MRI, Vessel wall magnetic resonance imaging; CTA, Computed tomography angiography; DSA, Digital subtraction angiography; DWI, Diffusion-weighted imaging; FLAIR, Fluid-attenuated inversion recovery; SWI, Susceptibility-weighted imaging; GCA, Giant cell arteritis; SPACE, Sampling perfection with application-optimized contrast using different flip angle evolution; T2W, T2-weighted; T1W, T1-weighted; IVWI, Intracranial vessel wall imaging; PD, Proton density; MRA, Magnetic resonance angiography; TIRM, Turbo inversion recovery magnitude; STIR, Short-tau inversion recovery; FOV, Field of view; SPAIR, Spectral attenuated inversion recovery; TIA, Transient ischemic attack; PACNS, Primary angiitis of the central nervous system; LSA, Lenticulostriate artery; NA, Not available

Study ID	Population	No of patients	No of lesions	Age mean ± SD	Study design	Study question	Imaging protocol	Reference standard modality
Mossa-Basha et al. (2015) [20]	Patients with suspicion of intracranial vasculopathy with luminal stenosis or irregularity	ICAD (21), RCVS (4), Vasculitis (4)	ICAD (81), RCVS (22), Vasculitis (15)	ICAD (53.8 ± 10.4), RCVS (43.2 ± 9.8), Vasculitis (34.2 ± 12.3)	Retrospective	Value of VW-MRI in differentiating intracranial vasculopathies	3T Siemens Trio: 3D TOF MRA, T1, T2 VW-MRI, pre/post-contrast	Clinico-radiological
Farag et al. (2020) [21]	Ischemic stroke patients within 2 weeks from onset	ICAD (11), Vasculitis (3), Moyamoya (2)	NA	From 18 to 80 years with a mean age of 49.38	Cross-sectional	Value of VW-MRI in the diagnosis of cerebrovascular diseases	3T VW-MRI, T1 pre/post-contrast, T2 fat sat	MRA
Liu et al. (2013) [22]	Patients presenting with symptoms and signs of recent, new-onset MCA territory infarcts	28	33	56.4 ± 12.8	Retrospective	Comparison of high-resolution MRI with CTA and DSA for the evaluation of MCA atherosclerotic steno-occlusive disease	CTA (Siemens SOMATOM (64) and Aquilion ONE, 0.5-0.6 mm slices, Ultravist 370), HR-MRI (3T GE HDXT, T1, T2, PD, STIR, 2 mm slices, 0.5 mm interval, no contrast), DSA (Femoral puncture, Ultravist, 1024x1024 matrix, 22 cm FOV)	DSA
Bai et al. (2018) [23]	Patients suspected of having intracranial atherosclerotic stenosis involving the MCA	62	80	From 45 to 80 years with a mean age of 61.3 years	Prospective	Value of 3D black-blood luminal angiography derived from high-resolution VW-MRI in detecting MCA stenosis	MRI (3T GE Discovery MR750, TOF-MRA, 2D fast spin-echo T2, 3D-Cube T1, 3D-Cube proton-density-weighted imaging), CTA (320-detector Aquilion ONE, 120 kV, 0.5 mm slices, Ultravist 370, 5 mL/s)	CTA
Tian et al. (2021) [24]	Patients with posterior circulation stroke and/or TIA who underwent DSA and 3D black-blood MRI within 3 months of the ischemic events, and within 4 weeks of each other	101	238	63 ± 10	Prospective	Assessment of intracranial atherosclerotic plaques using 3D black-blood MRI compared to 3D TOF MRA and DSA	DSA (Artis zee Biplane, 5-second acquisition, Ultravist, 200° rotation), MRI (3T Siemens Skyra, 3D TOF, 3D IVWI, pre/post-gadolinium contrast)	DSA
Bai et al. (2023) [25]	Patient with MCA atherosclerotic plaques	60	80 (37 culprit and 43 non-culprit)	NA	Prospective	Investigation of MCA plaque characteristics and LSA morphology associated with subcortical infarctions in LSA territories	7T, 3D TOF-MRA, 3D T1 SPACE	3D-TOF MRA
Kim et al. (2019) [26]	Patients with ICAD who underwent 3D TOF MRA within 2 months	17	286	From 42 to 91 years with a mean age of 60 years	Retrospective	Comparison of non-contrast VWI and 3-D TOF MRA for ICAD and plaque characterization within intracranial arteries	VWI (3D Proton Density SPACE/CUBE, 16 cm FOV, 3T with no contrast), 3D-TOF MRA (3T magnets, 0.6 mm slices, matrix 320x320, 18 cm FOV)	3D-TOF MRA
Tian et al. (2023) [27]	Patients with cerebral ischemic symptoms of the unilateral anterior circulation, with stenosis <50%, and underwent 3D HR-VW-MRI within 1 month of ischemic events	150	MCA 133 plaques (97 culprit and 36 non-culprit), ACA 3 plaques (3 culprit), ICA 14 Plaques (11 culprit and 3 non-culprit)	Culprit (58.5 ± 12.6), non-culprit (58.9 ± 10.6)	Retrospective	Analyzing the difference in quantitative features between culprit and non-culprit intracranial plaque without substantial stenosis using 3D HR-VW-MRI	Siemens Skyra 3T, 3D TOF MRA, HR-VWI, T2-weighted, T1-weighted SPACE, gadolinium	CTA or MRA
Fakih et al. (2020) [28]	Patients with acute stroke of cryptogenic origin, and nonspecific arterial changes on CTA, MRA, or DSA	34	153	58.9 ± 14.7	Prospective	Detection and quantification of symptomatic atherosclerotic plaques with HR-VWI in cryptogenic stroke	HR-VWI (GE MR950 7T, 3D T1 CUBE, T2 CUBE, TOF, susceptibility-weighted angiogram, post-contrast gadobutrol)	CTA, MRA or DSA
Teng et al. (2015) [29]	Patients presented with symptoms and signs of MCA territory ischemia or infarction	139 (112 symptomatic and 27 asymptomatic)	165 lesions (112 culprit and 53 non-culprit)	For symptomatic patients (56.8 ± 10.2), and for asymptomatic patients (58.6 ± 11.1)	Retrospective	Incremental value of HR-MRI for identifying culprit plaques in MCA atherosclerosis	HR-MRI (3T GE HDx: 3D TOF MRA, 2D fast spin-echo T2, T1, gadolinium-enhanced T1)	Clinico-radiological
Ryu et al. (2022) [30]	Patients diagnosed with VAD based on clinical and radiological findings, who underwent HR-VWI within 7 days of symptom onset and DSA within 2 weeks of HR-VWI	24	27	From 31 to 78 years with a mean age of 56.6	Retrospective	Comparing the diagnostic performance of HR-VWI with DSA for intracranial vertebral artery dissection	MRI (3T Achieva/VIDA, 3D HR-VWI, TOF-MRA, T1WI, proton density, CE T1WI, gadolinium), DSA (Philips Allura Xper FD, biplane, ICA/VA, 4-1 F/s, 15 F/s fluoroscopy, 30 F/s rotational, 84 kVp, 210 mAs)	Clinico-radiological
Sui et al. (2021) [31]	Patients with suspected unruptured intracranial VBDAs	49 (34 VBDA, 7 flow artifacts, 8 atherosclerotic plaques)	35 VBDA lesions	50 ± 18.5	Retrospective	Value of HR-VW-MRI for depicting the imaging features of unruptured intracranial VBDAs	Siemens MAGNETOM Trio, 3T, 3D TOF, T2W, T1W, 3D T1W SPACE, contrast-enhanced T1W, gadolinium	Clinico-radiological (DSA)
Ferlini et al. (2022) [32]	Patients with a diagnosis of central nervous system angitis	38 patients (36 meeting initial inclusion criteria + 2 identified from stroke unit)	NA	60.1 ± 12	Retrospective	Sensitivity and specificity of VW-MRI to diagnose central nervous system angiitis	3T VW-MRI with 3D-T1 black blood pre/post-gadolinium, FLAIR, DWI, T2, TOF	Clinico-radiological (DSA or MRA) - Cerebral biopsy - CSF analysis
Eiden et al. (2019) [33]	Patients with clinical suspicion of primary CNS vasculitis, large vessel CNS vasculopathy, or secondary involvement of intracranial arteries in systemic vasculitis	39	2D VWI: 37 (vasculitic) + 40 (non-vasculitic) = 77 lesions, 3D VWI: 36 (vasculitic) + 42 (non-vasculitic) = 78 lesions	From 14 to 77 years, with mean age equals 52.5 years	Prospective	Value of contrast-enhanced HR-MRI in patients with suspected vasculitis	3T MRI: DWI, 3D TOF MRA, 2D T1 TIRM, 3D T1 SPACE VWI, prohance contrast	Clinico-radiological - Cerebral biopsy
Destrebecq et al. (2020) [34]	Patients admitted to stroke unit for acute neurological deficits (majority of them presented with focal neurological deficits of sudden onset)	20	NA	59 ± 13	Retrospective	Value of intracranial VW-MRI in cryptogenic stroke and intracranial vasculitis	For parenchymal imaging (FLAIR, DWI, T1, T2, T2*, 3D-TOF, 3D-T1 MRA with contrast), for vessel wall (3T 3D turbo-spin-echo T1-weighted with black blood prepulse, pre- and post-gadolinium)	Clinico-radiological (DSA)
Karaman et al. (2021) [35]	Patients with clinical suspicion of PACNS who presented with new-onset ischemic events and had significant intracranial large vessel stenosis on DSA or MRA	23 (10 PACNS and 13 indeterminate for PACNS)	37	37.5 ± 11.5	Prospective	Diagnostic value of VW-MRI in the differentiation of primary angiitis of the central nervous system from other intracranial vasculopathies	3T, 3D T1 (0.8 mm), gadobutrol, TOF, T2W, FLAIR, DWI, SWI	Clinico-radiological (MRA or DSA)
Poillon et al. (2019) [36]	Patients aged over 50 years with suspicion of GCA based on clinico-radiological assessment	79 (GCA 51, non-GCA 28)	NA	75 ± 9.5	Prospective	Comparing the diagnostic accuracy of 3D versus 2D contrast-enhanced VW-MRI of extracranial and intracranial arteries in the diagnosis of GCA	3T Philips/GE, 2D/3D CE-VWI, gadobutrol, SPAIR/FatSat	Clinico-radiological + Temporal artery biopsy

Quantitative Results

Diagnostic accuracy of VW-MRI in intracranial vasculitis: Except for Poillon et al.’s study [36], the included studies reported good-to-excellent sensitivity (range: 0.67-0.96), acceptable-to-excellent specificity (range: 0.48-0.96), and a wide range of DOR (1.87-414) (Table 2).

**Table 2:** Summary statistics of diagnostic performance per included studies (vasculitis) Correlation between sensitivity and FPR = ρ = -0.371 (p-value: 0.497). FPR, False positive rate; DOR, Diagnostic odds ratio

Study ID	Sensitivity	Specificity	DOR
	(95% CI)	(95% CI)	(95% CI)
Ferlini et al. (2022) [32]	0.95 (0.83-0.99)	0.96 (0.86-0.99)	414 (55.59-3083.34)
Farag et al. (2020) [21]	0.88 (0.4-0.99)	0.96 (0.73-1)	189 (3.16-11318.26)
Eiden et al. (2019) [33]	0.67 (0.42-0.85)	0.48 (0.31-0.66)	1.87 (0.51-6.83)
Destrebecq et al. (2020) [34]	0.96 (0.7-1)	0.83 (0.63-0.94)	115 (5.42-2440.84)
Karaman et al. (2021) [35]	0.95 (0.77-0.99)	0.69 (0.44-0.86)	44 (4.55-425.72)
Poillon et al. (2019) [36]	0.2 (0.12-0.33)	0.98 (0.85-1)	14.42 (0.81-256.1)

**Figure 2:**
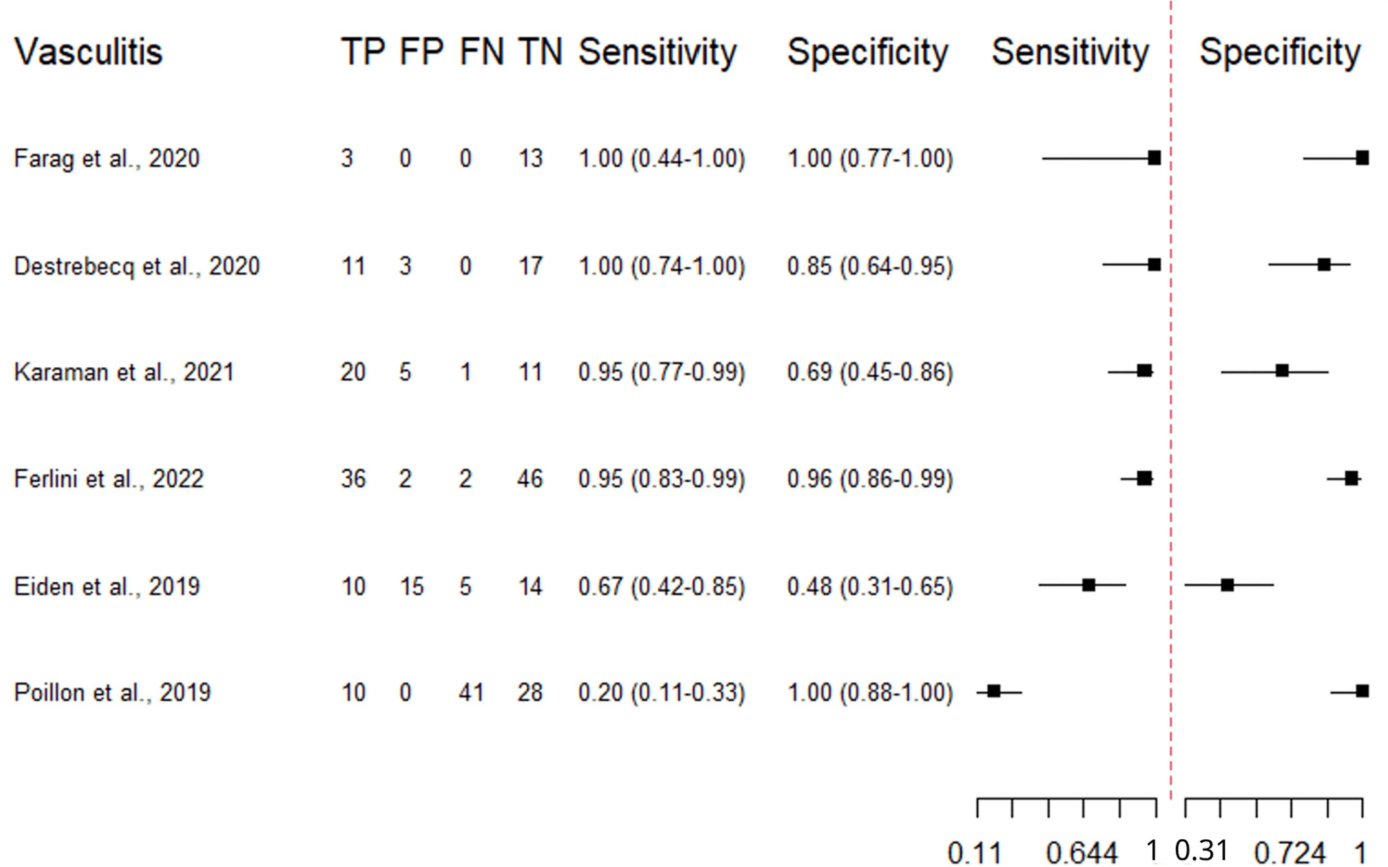
Descriptive Forest plot of the reported diagnostic performance (vasculitis) Each study’s sensitivity and specificity are shown with 95% confidence intervals [21,32-36]. TP, True positives; FP, False positives; TN, True negatives; FN, False negatives

**Figure 3:**
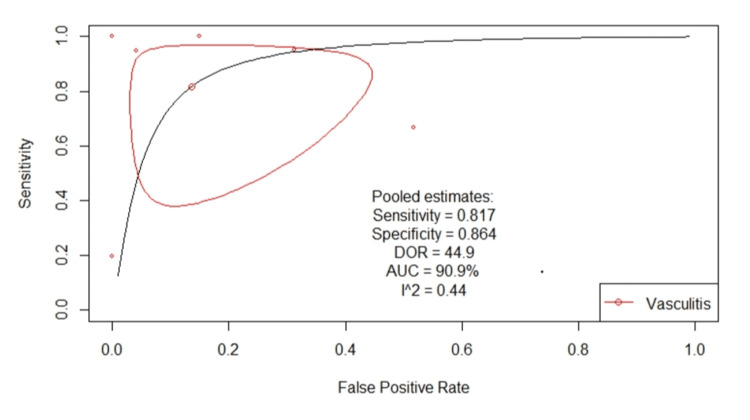
SROC curve for the pooled diagnostic performance of VW-MRI for intracranial vasculitis, estimated through bivariate modeling Analysis performed using a bivariate random-effects model (R package: mada); p < 0.05 was considered significant. SROC, Summary receiver operating characteristic; VW-MRI, Vessel wall magnetic resonance imaging; DOR, Diagnostic odds ratio; AUC, Area under the curve

**Figure 4:**
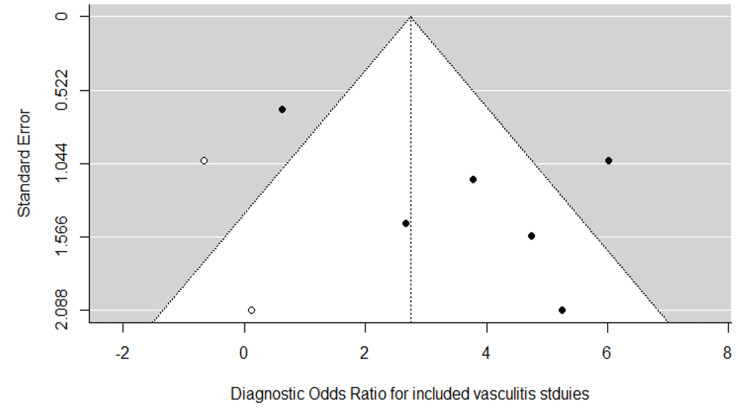
Funnel plot assessing potential publication bias among included vasculitis studies Analysis was performed using the trim-and-fill method, based on the log diagnostic odds ratio, within a bivariate random-effects model (R packages: mada and metafor). Statistical significance was set at p < 0.05.

Figure 2 shows a classical descriptive forest plot. Figure 3 shows the SROC curve for the pooled diagnostic performance of VW-MRI for intracranial vasculitis, which was estimated through bivariate modeling. The pooled sensitivity was 0.817 (95% CI: 0.476-0.957), and the pooled false positive rate was 0.136 (95% CI: 0.041-0.368), with a specificity of 0.864, a DOR of 44.9, and an AUC of 0.909. However, heterogeneity among the included studies was concerning (I²: 0.44), which may be due to the limited number of studies included (n = 6) and the limited sample size within some studies, which negatively affects the precision of the pooled estimates. The risk of publication bias was minimal (Figure 4).

**Figure 5:**
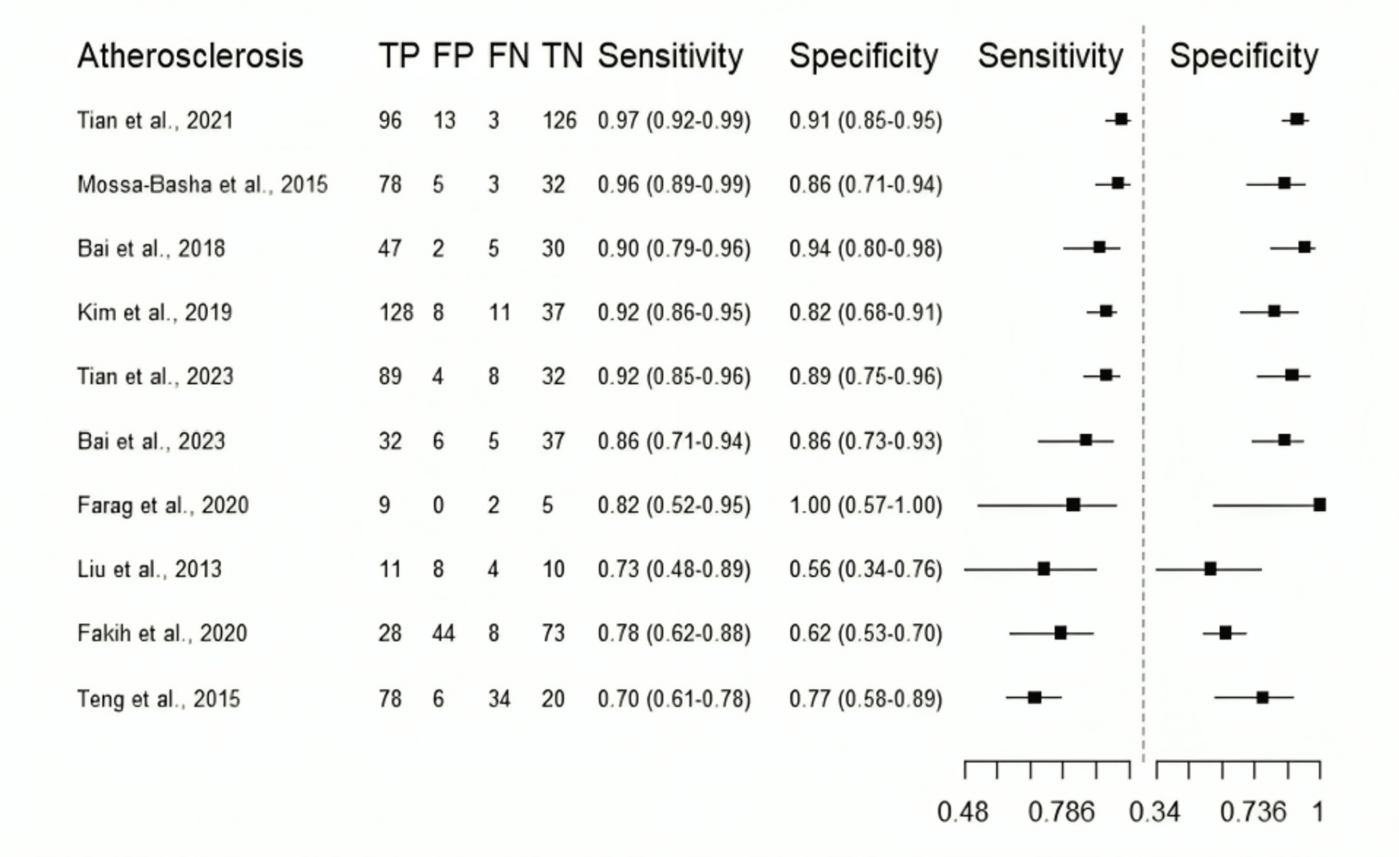
Descriptive Forest plot of the reported diagnostic performance (atherosclerosis) Each study’s sensitivity and specificity are shown with 95% confidence intervals [20-29]. TP, True positives; FP, False positives; TN, True negatives; FN, False negatives

**Figure 6:**
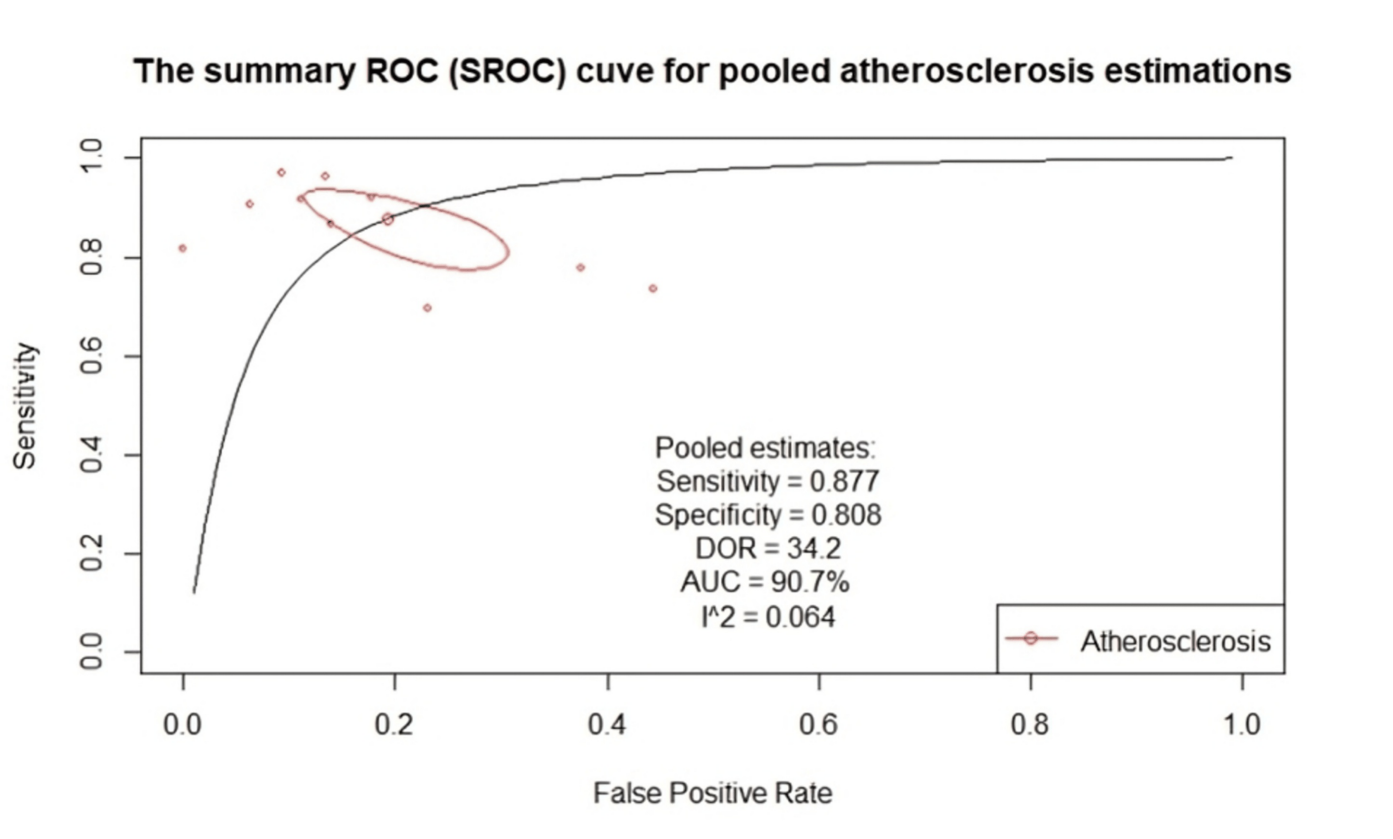
SROC curve for the pooled diagnostic performance estimated through bivariate modeling Analysis performed using a bivariate random-effects model (R package: mada); p < 0.05 considered significant. SROC, Summary receiver operating characteristic; DOR, Diagnostic odds ratio; AUC, Area under the curve

Diagnostic accuracy of VW-MRI for intracranial atherosclerosis: The included studies demonstrated good- to excellent sensitivity (range: 0.7-0.97), acceptable- to excellent specificity (range: 0.56-0.94), and a wide range of DORs (range: 5.81-310.15) (Table 3). Figure 5 shows a classical descriptive forest plot. From the forest plot, the highest performance was reported in Tian et al.’s study [24], with a sensitivity of 0.97, specificity of 0.91, and the highest DOR of 310.15. Figure 6 shows the pooled estimates from the bivariate model. The pooled sensitivity was 0.877 (95% CI: 0.799-0.927). This excellent performance was accompanied by a specificity of 0.808, a DOR of 34.2, and an AUC of 0.907. Additionally, the low level of heterogeneity noted (I²: 0.064) further indicates the reliability of our pooled estimates.

**Table 3:** Summary statistics of diagnostic performance per included studies (atherosclerosis) Correlation between sensitivity and FPR = ρ = 0.539 (p-value: 0.113). FPR, False positive rate; DOR, Diagnostic odds ratio

Study ID	Sensitivity (95% CI)	Specificity (95% CI)	DOR (95% CI)
Mossa-Basha et al. (2015) [20]	0.96 (0.9-0.99)	0.86 (0.72-0.94)	166.4 (37.53-737.83)
Farag et al. (2020) [21]	0.79 (0.51-0.93)	0.92 (0.52-0.99)	41.8 (1.68-1038.71)
Liu et al. (2013) [22]	0.73 (0.48-0.89)	0.56 (0.34-0.75)	3.44 (0.79-15.02)
Bai et al. (2018) [23]	0.9 (0.79-0.96)	0.94 (0.8-0.98)	141 (25.69-773.83)
Tian et al. (2021) [24]	0.97 (0.91-0.99)	0.91 (0.85-0.94)	310.15 (85.96-1119.04)
Kim et al. (2019) [26]	0.92 (0.86-0.96)	0.82 (0.69-0.91)	53.82 (20.17-143.6)
Bai et al. (2023) [25]	0.86 (0.72-0.94)	0.86 (0.73-0.93)	39.47 (11-141.61)
Tian et al. (2023) [27]	0.92 (0.85-0.96)	0.89 (0.75-0.96)	89 (25.08-315.78)
Fakih et al. (2020) [28]	0.78 (0.62-0.88)	0.62 (0.53-0.71)	5.81 (2.43-13.86)
Teng et al. (2015) [29]	0.7 (0.61-0.77)	0.77 (0.58-0.89)	7.65 (2.82-20.73)

**Figure 7:**
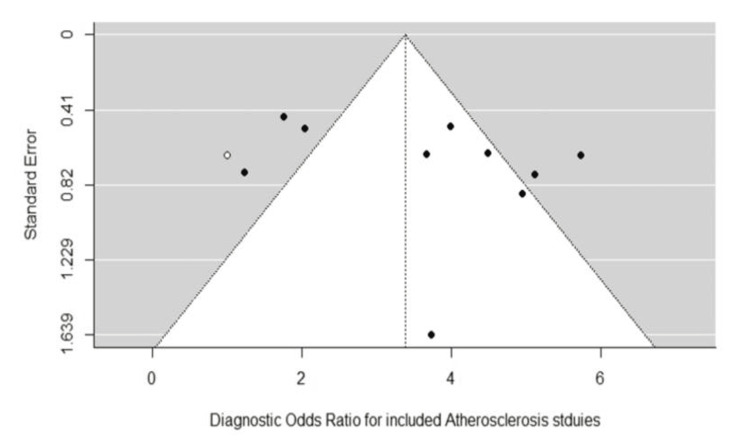
Funnel plot assessing potential publication bias among included atherosclerosis studies Analysis performed using the trim-and-fill method, based on the log diagnostic odds ratio, within a bivariate random-effects model (R packages: mada and metafor). Statistical significance was set at p < 0.05.

Table 4 shows the results of the subgroup analysis performed to detect any variation in the performance of our diagnostic test between the different outcomes. The 10 included studies had a total of four defined outcomes: detection of culprit vs. non-culprit (n = 4 studies), detection of >50% vessel stenosis (n = 3), detection of eccentric thickening (n = 2), and detection of luminal stenosis (n = 1; excluded from the current subgroup analysis). No significant difference was observed between the different outcome definitions (p > 0.05). The heterogeneity decreased to 0.001 (compared to 0.064 in Figure 5), indicating that the other outcome definitions explained some of the heterogeneity observed in the data. However, this association does not appear to influence diagnostic test performance metrics. These findings indicate that VW-MRI is a reliable tool for detecting radiological biomarkers, such as >50% vessel stenosis and vessel wall eccentric thickening, and that its accuracy in detecting culprit lesions is noteworthy. The risk of publication bias was minimal (Figure 7).

**Table 4:** Outcome-defined subgroup analysis of diagnostic performance in detecting atherosclerosis

Term	Estimate (95% CI)	Pr(>|z|)
[Sensitivity].(Intercept)	1.462 (0.738 to 2.185)	0.000
[Sensitivity].Outcome: Detection of >50% Stenosis	0.646 (-0.54 to 1.833)	0.286
[Sensitivity].Outcome: Eccentric thickening	0.899 (-0.545 to 2.344)	0.222
[FPR].(Intercept)	-1.194 (-1.907 to -0.482)	0.001
[FPR].Outcome: Detection of >50% Stenosis	-0.337 (-1.458 to 0.785)	0.556
[FPR].Outcome: Eccentric thickening	-0.361 (-1.852 to 1.13)	0.635
I^2	0.001	-

**Figure 8:**
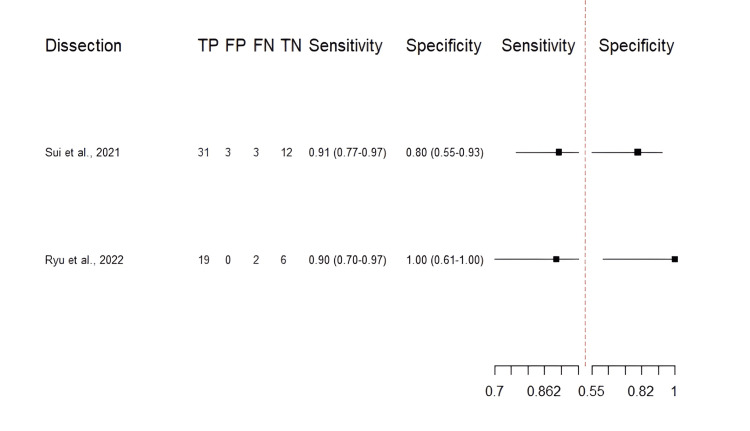
Descriptive Forest plot of the reported diagnostic performance (dissection) Each study’s sensitivity and specificity are shown with 95% confidence intervals [30,31]. TP, True positives; FP, False positives; TN, True negatives; FN, False negatives

**Figure 9:**
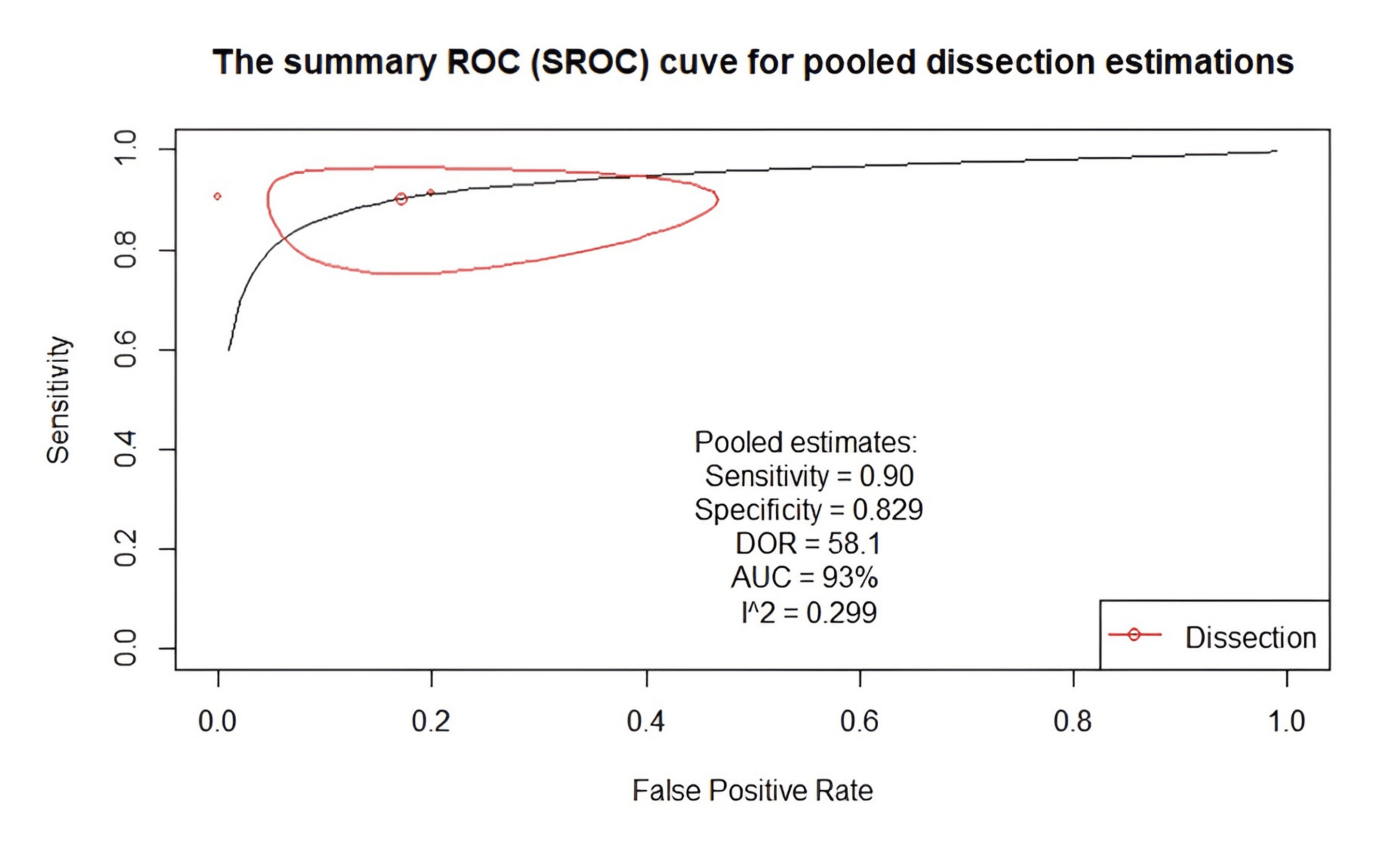
SROC curve for the pooled diagnostic performance estimated through bivariate modeling Analysis performed using a bivariate random-effects model (R package: mada); p < 0.05 considered significant. SROC, Summary receiver operating characteristic; DOR, Diagnostic odds ratio; AUC, Area under the curve

Diagnostic accuracy of VW-MRI for intracranial dissections: The included studies had excellent sensitivity (0.89-0.91), specificity (0.80-0.93), and DOR (41.3-101.4) (Table 5). Figure 8 shows a classical descriptive forest plot, and Figure 9 shows the SROC curve for the pooled diagnostic performance, estimated through bivariate modeling.

**Table 5:** Summary statistics of diagnostic performance per included studies (dissection) Correlation between sensitivity and FPR = ρ = -1 (p-value: 1) FPR, False positive rate; DOR, Diagnostic odds ratio

Study ID	Sensitivity (95% CI)	Specificity (95% CI)	DOR (95% CI)
Ryu et al. (2022) [30]	0.89 (0.69-0.96)	0.93 (0.56-0.99)	101.4 (4.29-2398.35)
Sui et al. (2021) [31]	0.91 (0.77-0.97)	0.8 (0.55-0.93)	41.33 (7.3-233.96)

The pooled sensitivity was 0.901 (95% CI: 0.791-0.956), with a specificity of 0.829, a DOR of 58.1, and an AUC of 0.93. Additionally, the low level of heterogeneity (I²: 0.299) further indicated the reliability of our pooled estimates. However, these results were derived from only two studies, both of which were small and at potential risk of bias. Therefore, these findings should be interpreted with extreme caution, and further high-quality, prospective studies are needed to validate the diagnostic accuracy of VW-MRI for intracranial dissections. A funnel plot was not applicable due to the limited number of included studies.

​​​*Quality Assessment*

The QUADAS tool demonstrated the high quality of the included studies. Table 6 shows the quality assessment questions, which are provided in the Appendix.

Discussion

The findings of this meta-analysis highlight the strong diagnostic performance of VW-MRI for various intracranial conditions. VW-MRI consistently demonstrated high sensitivity and specificity for intracranial vasculitis, atherosclerosis, and dissection, with excellent sensitivity, specificity, and AUC. We aimed to quantitatively pool the existing evidence on the diagnostic performance of VW-MRI. However, we believe that there are considerable gaps in this area, and that research is ongoing. VW-MRI has been used widely in clinical practice at many centers since the formation of a multidisciplinary study group to support the development and clinical implementation of VW-MRI by the American Society of Neuroradiology in 2012 [7].

VW-MRI can differentiate between various intracranial arterial pathologies by detecting specific patterns in the vessel walls. Despite some overlap, each pattern shows a degree of specificity. Tritanon et al. retrospectively reviewed 3T high-resolution vessel wall imaging studies of patients diagnosed with atherosclerotic plaques, vasculitis, and dissection, and differentiated between these conditions based on the patterns of involvement, wall enhancement, T1 and T2 signals, and specific patterns [37]. Their study showed concentric wall thickening in 84.6% of patients with vasculitis and eccentric wall thickening in 97% of patients with atherosclerosis and 92.3% of patients with dissection. Additionally, significant differences in wall enhancement and specific patterns were observed among the three conditions. These findings are consistent with those of previous reports [38-40]. Mossa-Basha et al. studied 118 stenotic intracranial lesions (81 ICAD, 22 reversible cerebral vasoconstriction syndrome, and 15 vasculitic lesions) and reported that ICAD showed significantly higher eccentric wall involvement (90.1%) compared with reversible cerebral vasoconstriction syndrome (8.2%) and vasculitic lesions (6.7%), and was more likely to have T2 hyperintensity (79% vs. 0%). Notable differences in the enhancement patterns were observed between the lesion types [20].

VW-MRI showed a remarkable ability to detect culprit intracranial plaque vs. non-culprit plaque using radiologic biomarkers obtained by VW-MRI, beyond luminal stenosis. Tian et al. recruited patients with cerebral ischemic symptoms of unilateral anterior circulation with non-stenotic intracranial atherosclerosis (<50%) [27]. They reported that PV and maximum wall thickness can be used to identify lesions responsible for acute ischemic stroke in patients with intracranial atherosclerosis, with sensitivities and specificities of 91% and 89%, respectively. Teng et al. reported that VW-MRI data, such as PB and MLA, in addition to stenosis, improved the diagnostic performance for detecting culprit lesions [29].

Bai et al. found that a combination of lenticulostriate artery origin involvement and plaque irregular surface, obtained by 7T VW-MRI, showed good performance in identifying culprit plaques, with sensitivities and specificities of 86.5% and 86.0%, respectively [25]. Furthermore, Fakih et al. reported that a contrast ratio ≥53 had a sensitivity of 78% and specificity of 62% [28].

VW-MRI can help identify vessel stenosis and lesion length more efficiently than other luminal angiographic imaging. Bai et al. compared the diagnostic performance of black-blood luminal angiography derived from 3D vessel wall imaging with time-of-flight MRA for detecting middle cerebral artery stenosis and reported that black-blood luminal angiography showed a significantly higher sensitivity for detecting severe stenosis (89.3% vs. 64.3%) [23]. The lesion length estimated on source images of vessel wall imaging was substantially greater than that measured by CTA and black-blood luminal angiography (p = 0.001 and p = 0.01). These findings are supported by other studies [24,26].

Intramural inflammatory cell infiltration in vasculitis can cause damage to the mural integrity, resulting in contrast uptake [41]. Therefore, concentric VWE has promising sensitivity and specificity as a radiological biomarker of intracranial vasculitis. Ferlini et al. reported that concentric VWE could efficiently identify patients affected by medium-sized vessel central nervous system vasculitis, with a specificity of 95% and sensitivity of 94% [32]. Other studies reported the same high sensitivity [21,34,35] and specificity [21,34,36]. However, Eiden et al. reported lower sensitivity and specificity (67% and 48%) [33]. Poillon et al. reported the lowest sensitivity value (20%), but with excellent specificity (100%) [36]. These findings are consistent with those of our study, which yielded high diagnostic accuracy: pooled sensitivity of 0.817, specificity of 0.864, and AUC of 0.909. As an advanced technique, VW-MRI acquisition and interpretation should be performed by experts familiar with the common sources of errors in interpreting intracranial VW-MRI examinations, including the challenges of differentiating between the causes of abnormal wall thickening and enhancement, and distinguishing these changes from commonly encountered artifacts. This is essential for the diagnostic confidence and performance of radiologists who interpret these examinations [4,42].

This study has some limitations that should be acknowledged. This study included a small number of studies per condition - particularly for dissections - with some studies also having small sample sizes, which may have limited the statistical power of our results. Additionally, the included studies were observational, with most being retrospective, which may introduce a potential source of bias. The most significant limitation of our study was the lack of a gold standard reference test and the heterogeneity of imaging modalities and imaging protocols used in each study, which may have introduced possible data heterogeneity and affected pooled estimates of diagnostic accuracy. However, this is an ongoing limitation in research in this area because the ideal method - pathology-imaging correlation studies - is not applicable. Although stratification by reference modality was considered, the limited number of studies within each subgroup prevented reliable comparative analysis. Future research should aim to standardize reference criteria and perform head-to-head assessments using DSA to validate VW-MRI findings. Finally, studies focusing on differentiating culprit from non-culprit plaques employed variable imaging parameters, which may have further contributed to heterogeneity in reported diagnostic performance. Nevertheless, our pooled results provide meaningful insights into the overall diagnostic performance of VW-MRI rather than reflecting the accuracy of a single imaging biomarker.

## Conclusions

This study highlights the high diagnostic performance of VW-MRI for differentiating intracranial vasculopathies (atherosclerosis, vasculitis, and dissection). VW-MRI is a valuable imaging modality that can provide a detailed characterization of intracranial vessel walls that cannot be achieved using traditional luminal-based imaging techniques. VW-MRI can detect specific radiologic biomarkers, enhancing its clinical utility in diagnosing and managing intracranial vasculopathy. VW-MRI has proven to be a more valuable tool for detecting vessel stenosis and culprit lesions using different parameters. Given the study’s limitations and heterogeneity, continued research and technological advancements are needed to further establish the role of VW-MRI in clinical decision-making for intracranial vascular diseases.

## Appendices

**Table 6:** QUADAS tool for quality assessment Q1: Was the spectrum of patients representative of the patients who will receive the test in practice? (Spectrum Bias) Q2: Were selection criteria clearly described? (Selection Bias) Q3: Is the reference standard likely to correctly classify the target condition? (Reference Standard Bias) Q4: Is the time period between reference standard and index test short enough to be reasonably sure that the target condition did not change between the two tests? (Temporal Bias) Q5: Did the whole sample or a random selection of the sample, receive verification using a reference standard of diagnosis? (Verification Bias) Q6: Did patients receive the same reference standard regardless of the index test result? (Verification Bias) Q7: Was the reference standard independent of the index test (i.e., the index test did not form part of the reference standard)? (Verification Bias) Q8: Was the execution of the index test described in sufficient detail to permit replication of the test? (Execution Bias) Q9: Was the execution of the reference standard described in sufficient detail to permit its replication? (Execution Bias) Q10: Were the index test results interpreted without knowledge of the results of the reference standard? (Verification Bias) Q11: Were the reference standard results interpreted without knowledge of the results of the index test? (Review Bias) Q12: Were the same clinical data available when test results were interpreted as would be available when the test is used in practice? (Clinical Applicability Bias) Q13: Were uninterpretable/intermediate test results reported? (Reporting Bias) Q14: Were withdrawals from the study explained? (Attrition Bias)

Study ID	Q1	Q2	Q3	Q4	Q5	Q6	Q7	Q8	Q9	Q10	Q11	Q12	Q13	Q14	Total score
Mossa-Basha et al. (2015) [20]	Yes	Yes	Yes	Unclear	Yes	Yes	Yes	Yes	Yes	Yes	Yes	Yes	No	Yes	12
Farag et al. (2020) [21]	Yes	Yes	Yes	Yes	Yes	Yes	Yes	Yes	Yes	Unclear	Unclear	Yes	No	Yes	11
Liu et al. (2013) [22]	Yes	Yes	Yes	Yes	Yes	Yes	Yes	Yes	Yes	Yes	Unclear	Yes	No	Yes	12
Bai et al. (2018) [23]	Yes	Yes	Yes	Yes	Yes	Yes	Yes	Yes	Yes	Unclear	Unclear	Yes	Yes	Yes	12
Tian et al. (2021) [24]	Yes	Yes	Yes	Yes	Yes	Yes	Yes	Yes	Yes	Unclear	Unclear	Yes	Yes	Yes	12
Bai et al. (2023) [25]	Yes	Yes	Yes	Unclear	Yes	Yes	Yea	Yes	Yes	Unclear	Yes	Yes	Unclear	Yes	11
Kim et al. (2019) [26]	Yes	Yes	Yes	Yes	Yes	Yes	Yes	Yes	Yes	Unclear	Unclear	Yes	Yes	Yes	12
Tian et al. (2023) [27]	Yes	Yes	Yes	Yes	Yes	Yes	Yes	Yes	Yes	Unclear	Unclear	Yes	No	Yes	11
Fakih et al. (2020) [28]	Yes	Yes	Yes	No	Yes	Yes	Yes	Yes	Yes	No	Yes	Yes	No	Yes	11
Teng et al. (2015) [29]	Yes	Yes	Yes	Unclear	Yes	Yes	Yes	Yes	Yes	Yes	Yes	Yes	No	No	11
Ryu et al. (2022) [30]	No	Yes	Yes	Yes	Yes	Yes	Yes	Yes	Yes	Yes	Yes	Yes	Unclear	No	11
Sui et al. (2021) [31]	No	Yes	Yes	Yes	Yes	Yes	Yes	Yes	Yes	Yes	Yes	Yes	No	No	11
Ferlini et al. (2022) [32]	Yes	Yes	Yes	Unclear	Yes	Yes	Yes	Yes	Yes	Unclear	Yes	Yes	No	Yes	11
Eiden et al. (2019) [33]	Yes	Yes	Yes	Unclear	Yes	Yes	Yes	Yes	Yes	Yes	Yes	Yes	Yes	Unclear	12
Destrebecq et al. (2020) [34]	Yes	Yes	Yes	Yes	Yes	Yes	Yes	Yes	Yes	Yes	Yes	Yes	No	No	12
Karaman et al. (2021) [35]	Yes	Yes	Yes	Yes	Yes	Yes	Yes	Yes	Yes	Yes	Yes	Yes	Yes	No	13
Poillon et al. (2019) [36]	Yes	Yes	Yes	Unclear	Yes	Yes	Yes	Yes	Yes	Yes	Yes	Yes	Yes	Yes	13
